# Are uroflowmetry and post - void residual urine tests necessary in children with primary nocturnal enuresis?

**DOI:** 10.1590/S1677-5538.IBJU.2017.0464

**Published:** 2018

**Authors:** Shang-Jen Chang, Stephen Shei-Dei Yang

**Affiliations:** 1 Division of Urology, Taipei Tzu Chi Hospital, Buddhist Tzu Chi Medical Foundation, New Taipei City, Taiwan, and School of Medicine, Buddhist Tzu Chi University, Hualien, Taiwan; Buddhist Tzu Chi University, School of Medicine, Hualien, Taiwan

**Keywords:** Urinary Bladder, Neurogenic, Enuresis, Child

## Abstract

**Objectives:**

To examine the benefits of repetitive uroflowmetry and post void residual urine (PVR) tests in children with primary nocturnal enuresis (PNE).

**Material and methods:**

Children aged ≥6 years with PNE who visited our clinics for management of enuresis were included for study. Patients were requested to complete a questionnaire including baseline characteristics and Dysfunctional Voiding Symptom Score (DVSS), 2-day bladder diary, and Rome III criteria for constipation. Two uroflowmetry and PVR tests were requested. Children with congenital or neurogenic genitourinary tract disorders were excluded. All children underwent urotherapy and desmopressin combined with anticholinergics or laxatives if indicated. The definition of abnormal flow patterns (≥1 abnormal), elevated PVR (≥1 abnormal), small maximal voided volume (MVV), nocturnal polyuria (NP) and response to treatment complied with the ICCS standardization document. Kaplan-Meier survival analysis and Cox proportional-hazards regression tests were used to evaluate the predictors of response.

**Results:**

In total, 100 children aged 8.5±2.3 years were enrolled for study (M: F=66:34) with 7.3±7.4 months of follow-up. Poor correlation was observed between DVSS/small MVV and PVR (p>0.05). Univariate analysis revealed that elevated PVR is associated with significantly less hazard of complete response to medical treatment (HR: 0.52, p=0.03), while not significantly associated with abnormal flow patterns, NP, constipation or small MVV. Multivariate analysis revealed that only elevated PVR (HR 0.30, 95% CI 0.12-0.80) and NP (HR 2.8, 95% CI 1.10-7.28) were significant predictors for complete response.

**Conclusions:**

In managing pediatric enuresis, elevated PVR is a significant predictor for lower chance of complete response to treatment whether they had high DVSS or not.

## INTRODUCTION

Nocturnal enuresis is defined as intermittent incontinence of urine during sleeping, with prevalence of 16.1% and 10.1% at age of 5 and 7 years, respectively, and decreasing as age increases (1, 2). The etiology could be attributed to nocturnal polyuria, small functional bladder capacity, arousal problem, or a mixture of the etiologies (3). Enuretic children can be classified as non-monosymptomatic (NMNE) or monosymptomatic nocturnal enuresis (MNE), depending on whether the child has daytime lower urinary tract symptoms or not (4). The first-line management of NMNE is to manage constipation, lower urinary tract symptoms and comorbid behavioral disorders. For enuresis symptoms in NMNE and MNE, the management included enuresis alarm as behavioral therapy and desmopressin. The predictive factors for response to medical treatment include age, disease severity, nocturnal diuresis and functional bladder capacity (3). Elevated post-void residual (PVR) was regarded as an important poor prognostic factor in NMNE while the prevalence rate of elevated PVR is assumed to be low in children with MNE (5); the International Children's Continence Society (ICCS), therefore, does not suggest bladder ultrasound as a preliminary screening diagnostic tool (5, 6). In our clinics, we routinely screen all enuretic children with two sets of uroflowmetry and post-void residual urine tests (PVR). Recent studies have stated that PVR was the only non-invasive diagnostic test to predict the treatment outcome in children with non-neurogenic lower urinary tract dysfunction (7). Elevated PVR may be associated with lower urinary tract dysfunction that cannot be identified by other noninvasive tests. Recently, the ICCS has adopted new criteria for defining elevated PVR in children (8, 9). Therefore, we retrospectively review the charts of children visiting our clinics for primary nocturnal enuresis to evaluate whether abnormal uroflowmetry and elevated PVR results are predictive of treatment response to urotherapy and medical treatment.

## MATERIALS AND METHODS

The study was approved by the institutional review board of our hospital and was designed as a retrospective review of the treatment response of primary nocturnal enuresis in toilet-trained children aged 6 years or older presenting to our pediatric urologic clinic. The parameters used for analysis included age, gender, and questionnaires. One parent who primarily cares for the child was asked to fill out the questionnaire, which included a 7-day enuresis diary before medical treatment, 48 hour bladder diary, Rome III questionnaire for functional constipation (10), and dysfunctional voiding symptom score (DVSS, 10 items, each scored 0-3) (11, 12). Children with neurological anomalies, neurogenic bladder or congenital genitourinary anomalies were excluded. Each child was asked to undergo a non-invasive diagnostic workup for lower urinary tract function with two sets of uroflowmetry and PVR tests on the same day. Only uroflowmetry curves with a voided volume of >50 mL were considered to be relevant for interpretation (13). PVR was calculated using the equation of height × width × depth × 0.52 (14). Maximal voided volume (MVV), daily voiding frequency, and nighttime diuresis volume were determined based on the 2-day bladder diary. MVV included the first void in the morning. Daily voiding frequency was determined as the average voiding frequency of the two days of records. Expected bladder capacity (EBC) was defined as (age in years x 30 + 30) mL (4). Nocturnal polyuria was defined as nighttime urine output >130% EBC (4). For children aged 6 and ≥7 years, elevated PVR in milliliters was defined as >20 and >10 mL, respectively (15). All enuretic children underwent urotherapy after the evaluations and were asked to have fluid restriction 1 to 2 hours before going to bed. Children with daytime urgency and small MVV were given oxybutynin and constipation was managed with magnesium oxide. As the alarm therapy was not covered by our insurance system and there existed no approved alarm system by Ministry of Health and Welfare in Taiwan, all children underwent desmopressin therapy with dosages of 0.1 to 0.4mg, and a structured withdrawal strategy was used. Follow-up data included the types of medication taken, enuretic episodes per week after treatment, and dryness status. Complete response to treatment was defined as a reduction of enuresis episodes by more than 90% in the past month during the follow-up, without recurrence (4).

### Statistics analysis

Data was expressed as mean±standard deviation and analyzed with MedCalc Statistical Software version 16.8 (MedCalc Software^®^, Ostend, Belgium; https://www.medcalc.org; 2016). Demographic and voiding parameters were compared via an independent sample t test (continuous demographic variables), a χ^2^ test (nominal data), and a Mann-Whitney U test (ordinal data). The log-rank test was used to compare the complete response between each parameter. The multivariate Cox proportional hazards regression with stepwise selection (enter and remove variable if p>0.05 and >0.1, respectively) was used to evaluate the predictive factors, including age (years), gender (boys vs. girls), constipation defined by Rome III (yes vs. no), DVSS (>6 vs. ≤6 (12)), nocturnal polyuria (nighttime urine amount >130% or ≦130% EBC), small functional bladder capacity (MVV≥65% vs. <65% EBC (4)), abnormal flow patterns (both bell vs. ≥1 non-bell), elevated PVR (≥1 abnormal vs. no abnormal) for complete response of enuresis. A Kolmogorov-type supremum test was used to assess the proportional hazards assumption. Correlation between DVSS and PVR were evaluated with Spearman's correlation. A p value of <0.05 was considered to be statistically significant.

## RESULTS

Between 2005 and 2013, 100 children with a mean age of 8.5±2.3 years that visited our clinics for management of primary enuresis were enrolled for study (M: F=66:34). Table-1 summarizes the demographic data and results of the medical tests for these children. There were no significant differences in age, follow-up period, maximal voided volume or daily voiding frequency between genders, except that girls had higher peak flow rate. There was no difference between the results of the first and second uroflowmetry and PVR tests in terms of voided volume (139.0±93.9 vs. 146.9±82.6 p=0.32), peak flow rate (17.9±7.1 vs. 18.9±8.3; p=0.11) or PVR (14.7±19.2 vs. 12.6±11.2; p=0.28). Among these children, the prevalence of constipation defined with Rome III, high DVSS (>6 on total score (12)), nocturnal polyuria (>130% EBC), small functional bladder capacity (MVV <65% EBC), ≥1 abnormal flow patterns, and ≥1 elevated PVR, were 20%, 38%, 19%, 18%, 42%, and 54% respectively.

**Table 1:** Baseline characteristics of the enrolled patients.

	Girls (n=34)	Boys (n=66)	p-value
Age (years)	9.1±2.6	8.2±2.0	0.05
Height (cm)	135.9±15.8	132.1±15.7	0.37
Weight (kg)	30.8±10.1	29.1±11.3	0.54
BMI (kg/m^2^)	17.0±3.2	16.7±3.3	0.66
Enuresis episode/week	5.7±1.7	5.9±1.8	0.56
MVV (mL)	91.7±65.6	108.3±85.4	0.23
Urine frequency 1^st^/2^nd^ (times/day)	7.8±3.2 / 6.8±3.9	8.4±2.8 / 8.1±2.8	0.37/0.10
Daily urine amount 1^st^/2^nd^ (mL)	753.8±333.0 / 904.4 ± 342.3	932.3±497.1 / 925.7±413.6	0.13/0.84
DVSS (score)	4.7±4.1	4.7±3.9	0.96
Peak flow rate 1^st^/2^nd^ (mL/sec)	21.2±7.8 / 19.4±7.6	17.5±6.7 / 18.8±8.5	0.02/0.81
PVR1^st^/2^nd^ (mL)	11.8±9.6 / 10.3±5.7	14.8±20.4 / 13.3±12.3	0.46/0.33

### Correlation of DVSS and small functional bladder capacity with PVR

The comparison of first and second PVRs in children with and without high DVSS was 13.4±18.3 vs. 14.5±18.7 (p=0.33) and 12.0±10.5 vs. 12.1±11.1 (p=0.94), respectively. Poorly correlation between PVR and DVSS (correlation coefficient: −0.01, p=0.94) was observed among these enuretic children. The comparison of first and second PVRs in children with and without small functional bladder capacity was 13.1 ± 19.2 vs. 14.6±16.5 (p=0.18) and 12.5±11.8 vs. 12.8±10.7 (p=0.97), respectively.

### Predictive factors for complete response of enuresis

**Figure 1:**
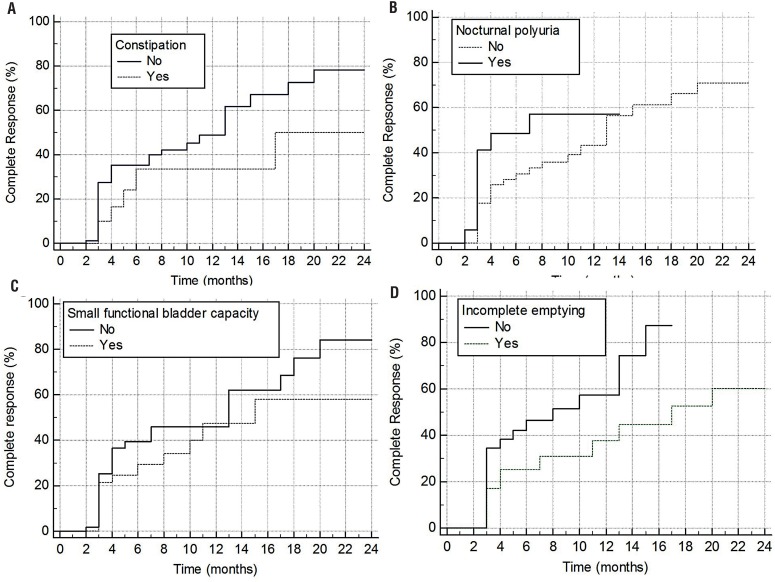
Comparison of complete response to desmopressin in enuretic children with and without A) constipation, B) Nocturnal polyuria, c) Small functional bladder capacity, D) Incomplete bladder emptying.

Univariate analysis showed that older age (HR: 0.88, 95CI: 0.75-1.02), boy gender (HR: 1.34, 95CI: 0.72-2.51), constipation (HR: 0.54, 95CI: 0.26-1.09, Figure-1A), high DVSS (HR: 1.08, 95CI:0.54-2.15), nocturnal polyuria (HR: 1.71, 95CI: 0.71-4.14, Figure-1B), small functional bladder capacity (HR: 0.92, 95CI: 0.40-2.15, Figure-1C), ≥1 abnormal flow patterns (HR: 0.87, 95CI: 0.44-1.73) were not associated with complete response, while ≥1 elevated PVR (HR: 052 95CI:0.27-0.98, p=0.03, Figure-1D) was statistically associated with complete response.

Multivariate cox proportional hazards regression revealed that only ≥1 elevated PVR (HR 0.30, 95% CI 0.12-0.80) and nocturnal polyuria (HR 2.8, 95% CI 1.10-7.28) were significant predictors for response of enuresis to treatment, while older age, boy gender, high DVSS, small functional bladder capacity, constipation and ≥1 abnormal flow patterns did not have a significant impact on response of enuresis to treatment.

## DISCUSSION

This is the first study that confirms the diagnostic role of PVR tests in predicting the response of primary enuretic children to treatment. Enuretic children with at least one elevated PVR are at significantly less risk (HR 0.30, 95% CI 0.12-0.80) of having complete response to urotherapy and medical treatment. Previous studies evaluating predictive factors for response focused on small bladder capacity (16), nocturnal polyuria (17, 18) and arousal problems (19). Few studies have addressed issues of lower urinary tract dysfunction, and the studies have mainly investigated the role of bladder wall thickness. In children with LUT dysfunction, PVR was one of the most important factors for diagnosing LUT dysfunction and monitoring treatment response (7). Elevated PVR may suggest some type of lower urinary tract dysfunction that cannot be identified by uroflowmetry and other noninvasive urodynamic studies. The ICCS does not recommend that children with enuresis receive routine evaluation of uroflowmetry or PVR tests (20, 21). Our study revealed poor correlation between DVSS/small MVV and PVR. Based on the significant predictive ability and on the fact that about half of enuretic children studied (54%) had at least one elevated PVR on current ICCS standards, we suggest that all children visiting clinics for primary nocturnal enuresis be evaluated with PVR tests whether they had high DVSS or not.

Cayan et al. (5) evaluated children with and without monosymptomatic enuresis, using uroflowmetry and PVR tests, and the authors concluded that monosymptomatic enuretic children did not have significantly higher PVRs compared with the control (5). The PVRs were 14.5±20.5 (n=48), 31.6±36.8 (n=40) and 19.8±26.4 (n=18) mL in enuretic children of variable age groups, respectively, compared to 6.4±9.9 (n=21, p=0.09), 23.7±30.4 (n=26, p=0.364), and 7.3±10.1 (n=10, p=0.08) mL in non-enuretic controls. There was a clear trend towards higher PVR in enuretic children; however, the difference was not statistically significant due to small sample size. In 2014, the ICCS adopted a new nomogram for defining elevated PVR (8). The PVR nomogram was established from data of normal healthy children (15). The results of the present study now confirm that the standard can be used to identify enuretic children who are less likely to respond to medical treatment. The new standards for the PVR nomogram were also used to predict the probability of recurrent urinary tract infection in children (22) and resolution of lower urinary tract dysfunction (7). Bladder wall thickness is the parameter of bladder dysfunction most commonly investigated to evaluate pediatric lower urinary tract dysfunction (23). However, the main drawback of bladder wall thickness lies in that wide inter-observer variability has been observed, and bladder volume greatly affects bladder wall thickness, which would compromise predictive ability.

Therefore, only some specific centers routinely measure bladder wall thickness. Unlike bladder wall thickness, the PVR test is widely adopted in physician's daily clinical practice for screening of lower urinary tract dysfunction and monitor treatment response. In enuretic children with elevated PVR, combined treatment with biofeedback and alpha blockers may help improve the treatment outcome (20).

The results of the current study show that 19% of children had nocturnal polyuria, and multivariate analysis revealed that children with nocturnal polyuria had a significantly higher chance for complete response. The results were in line with previous studies that showed that children with NP benefit more through desmopressin therapy, because the effect of the desmopressin is to suppress nighttime diuresis (17).

Practical consensus guidelines for the management of enuresis suggest that small for age bladder volume is associated with a lower rate of response to desmopressin and a higher response to enuresis alarm (3). Our study did not find a significant association of low functional bladder capacity (MVV<65%) with response to medical treatment in univariate (HR: 0.92, 95% CI: 0.40-2.15) and multivariate analysis. The possible explanations are incompleteness of 48 hour bladder diary, only a small proportion of children (18%) having small functional bladder capacity, and the combination therapy with oxybutynin.

DVSS is a validated symptom score used for screening children suspected of having lower urinary tract dysfunction (11). In our previous study, we found that children with a DVSS of >6 points were at higher risk of having dysfunctional voiding without gender difference (12). However, children with high DVSS were not significantly at greater risk of having poor response, which may be explained by these children presented with enuresis, while not daytime lower urinary tract symptoms, and small number of participants enrolled.

The pathophysiology for the impact of constipation on lower urinary tract dysfunction could be explained in that urinary bladder and rectum share common nerve innervations (24). Second, chronic constipation and rectal distention with stool may lead to external anal sphincter and pelvic floor muscle overactivity that lead to bladder dysfunction (24). Constipation diagnosed via the Rome III criteria was not significantly associated with the poor response in our study either (HR: 0.54, 95CI: 0.26-1.09). The small sample size and regular management of constipation at our clinics may attribute to the non-significance.

The major limitations of the study lie in that it is a retrospective review with a small sample size of patients enrolled from one institution. Larger-scale observations with this new PVR nomogram are required to further consolidate the role of PVR in predicting response to urotherapy and medical treatment. However, the PVR test is a significantly independent predictor for response, in addition to nocturnal polyuria. Despite the small sample size, elevated PVR clearly played a significantly diagnostic role. Second, as these children were referred from pediatric clinics for further management, most children had higher number of wet nights compared with other studies, and therefore the prevalence rate of PVR may be higher among them, though these children had not received medical treatment before. The major strength of our study is that each enrolled child had two sets of uroflowmetry and PVR tests. As such, we were able to identify the difference between repetitive elevated PVR and one elevated PVR on the response of enuresis to urotherapy and medical treatment.

## CONCLUSIONS

In summary, our retrospective review confirmed that elevated PVR in the newly published PVR nomograms predicted poor response of enuretic children with or without high DVSS to treatment.
